# The First Reported Outbreak of Chikungunya in the U.S. Virgin Islands, 2014–2015

**DOI:** 10.4269/ajtmh.16-0288

**Published:** 2016-10-05

**Authors:** Leora R. Feldstein, Esther M. Ellis, Ali Rowhani-Rahbar, M. Elizabeth Halloran, Brett R. Ellis

**Affiliations:** 1 Department of Epidemiology, School of Public Health, University of Washington, Seattle, Washington; 2 Vaccine and Infectious Disease Division, Fred Hutchinson Cancer Research Center, Seattle, Washington; 3 U.S. Virgin Islands Department of Health, Saint Croix, U.S. Virgin Islands; 4 Center for Inference and Dynamics of Infectious Diseases, Fred Hutchinson Cancer Research Center, Seattle, Washington; 5 Department of Biostatistics, School of Public Health, University of Washington, Seattle, Washington

## Abstract

The chikungunya virus (CHIKV) epidemic in the Americas is of significant public health importance due to the lack of effective control and prevention strategies, severe disease morbidity among susceptible populations, and potential for persistent arthralgia and long-term impaired physical functionality. Using surveillance data of suspected CHIKV cases, we describe the first reported outbreak in the U.S. Virgin Islands. CHIKV incidence was highest among individuals aged 55–64 years (13.1 cases per 1,000 population) and lowest among individuals aged 0–14 years (1.8 cases per 1,000 population). Incidence was higher among women compared to men (6.6 and 5.0 cases per 1,000 population, respectively). More than half of reported laboratory-positive cases experienced fever lasting 2–7 days, chills/rigor, myalgia, anorexia, and headache. No clinical symptoms apart from the suspected case definition of fever ≥ 38°C and arthralgia were significantly associated with being a reported laboratory-positive case. These results contribute to our knowledge of demographic risk factors and clinical manifestations of CHIKV disease and may aid in mitigating future CHIKV outbreaks in the Caribbean.

## Introduction

Chikungunya virus (CHIKV), an emerging alphavirus transmitted by the *Aedes* (*Stegomyia*) *aegypti* and *Aedes* (*Stegomyia*) *albopictus* mosquitoes, was introduced into the Americas in December 2013.^1^ As of April 2016, almost 2 million suspected or confirmed cases have been reported in 45 different countries in the Caribbean, Central, South, and North America.^1^ Acute symptoms of the virus, which include high fever, severe polyarthralgia, headache, and myalgia, often resolve within 7–10 days.^2^–^4^ However, up to 79% of cases from previous outbreaks in the Indian Ocean Basin, including American and European travelers, have reported persistent arthralgia, resulting in decreased quality of life for months after initial infection.^3^–^7^ Currently, there is no antiviral treatment or vaccine for the infection, there are no effective therapeutics for chronic symptoms, and public health prevention measures, such as vector control, have proven insufficient in preventing its spread.^2^,^8^

Between 1952 and 2000, CHIKV outbreaks had been reported in many Asian and African countries, typically with interepidemic periods of approximately 10 years.^9^ However, beginning in 2001, outbreaks began to occur yearly in Asia, Africa, Oceania, and Europe.^8^,^9^ In December 2013, the first case of CHIKV in the Americas was confirmed on the Caribbean island of Saint Martin.^10^ The U.S. Virgin Islands (USVI), one of the many regions in the Caribbean affected by the epidemic, identified four imported cases of CHIKV in January and February 2014. On June 6, 2014, the USVI Department of Health (DOH) identified the first locally acquired case of CHIKV on the island of Saint Thomas. In response to the initial cases of CHIKV, the USVI DOH worked in collaboration with the Centers for Disease Control and Prevention to establish and strengthen surveillance and diagnostic capacity for CHIKV and acute febrile illness, to educate healthcare providers and the public regarding CHIKV disease, and to provide recommendations for vector control and other mitigation efforts. Despite the swift response, almost 2,000 suspected cases of CHIKV were reported in the USVI (population = 103,574).^11^ The last laboratory-confirmed case was reported on February 23, 2015, and the last suspected case was reported on April 6, 2015.

The current CHIKV epidemic in the Americas is of significant public health importance due to the lack of sustainable and effective control and prevention strategies, the severe disease morbidity associated with a fully susceptible population, and the potential for persistent arthralgia leading to long-term impaired physical functionality of infected individuals.^12^,^13^ In addition, while the USVI has a total population of only 103,574, the territory receives almost 3 million visitors per year by air travel and cruise ship, which could further contribute to global CHIKV transmission.^11^,^14^,^15^

A detailed description of demographic information, clinical manifestations, and potential risk factors of laboratory-positive cases compared with laboratory-negative suspected cases, is essential to improving early identification of disease transmission for inevitable future outbreaks. In the present investigation, we describe the clinical epidemiology of the first CHIKV outbreak in the USVI during 2014–2015, as well as demographic risk factors associated with symptomatic CHIKV infection.

## Methods

### Study setting and subjects.

The three main islands of the USVI are Saint Thomas, Saint Croix, and Saint John with population sizes of 50,260, 49,255, and 4,059 people, respectively.^11^ Once the first confirmed case of CHIKV was recognized on June 6, 2014, all healthcare providers in the USVI were required to report suspected CHIKV cases to the USVI DOH using a standardized report form. As a result, residents of the USVI, who attended any of the three public hospitals or any public or private healthcare facility on Saint John, Saint Thomas, or Saint Croix and met the definition of a suspected CHIKV case, were captured by the USVI DOH surveillance system. The USVI DOH defined a suspected case of CHIKV as a resident of any age with acute onset of fever (≥ 38°C) and severe arthralgia or arthritis not explained by another medical condition. No active surveillance was conducted in the USVI during the outbreak; therefore, the sample used for this analysis is one of convenience and excludes individuals who were infected with CHIKV but did not seek health care.

### Data collection.

The data provided by the USVI DOH were deidentified, and each individual was represented by a unique reference identification code. The following information was collected using a standardized questionnaire for all suspected cases: age, sex, clinical symptoms, international travel 14 days before onset of illness, and contact with recently ill household members. A laboratory-positive case was defined as a suspected case with either: 1) isolation of CHIKV or demonstration of CHIKV nucleic acid in blood using reverse transcription polymerase chain reaction (RT-PCR) or 2) CHIKV-specific IgM antibodies in serum using enzyme-linked immunosorbent assay and CHIKV-specific neutralizing antibodies using plaque reduction neutralization test with a 90% plaque reduction cutoff.^16^,^17^ Individuals were confirmed negative if RT-PCR did not detect CHIKV nucleic acid in blood within the first 5 days of illness onset, or if individuals had no evidence of CHIKV-specific IgM antibodies in serum after the first 5 days of illness onset.^11^,^14^,^15^

### Study design and analysis.

The investigation is a cross-sectional study, examining the demographic and clinical differences between laboratory-positive CHIKV cases and laboratory-negative suspected cases. Descriptive statistics were used to summarize and compare these data. Reported CHIKV cases per 1,000 population were calculated by island, age category, and gender during the 2014–2015 outbreak. We generated an epidemic curve of laboratory-positive CHIKV cases per 1,000 island population by month. All data analyses were conducted using STATA 12 (StataCorp LP, College Station, TX).^19^

### Demographic characteristics.

To examine the association between CHIKV disease and individual demographic risk factors including: age, gender, contact with a recently ill household member, prior travel, and pregnancy status, prevalence ratios were calculated using Poisson regression with robust variance estimators.^20^ These risk factors were first examined separately and then together in a multivariate model.

### Clinical manifestations.

To determine additional clinical manifestations most strongly associated with CHIKV disease other than fever ≥ 38°C and arthralgia/arthritis, prevalence ratios were calculated for each symptom separately and together in a multivariate model using Poisson regression with robust variance estimators.^20^

## Results

**Figure 1.**
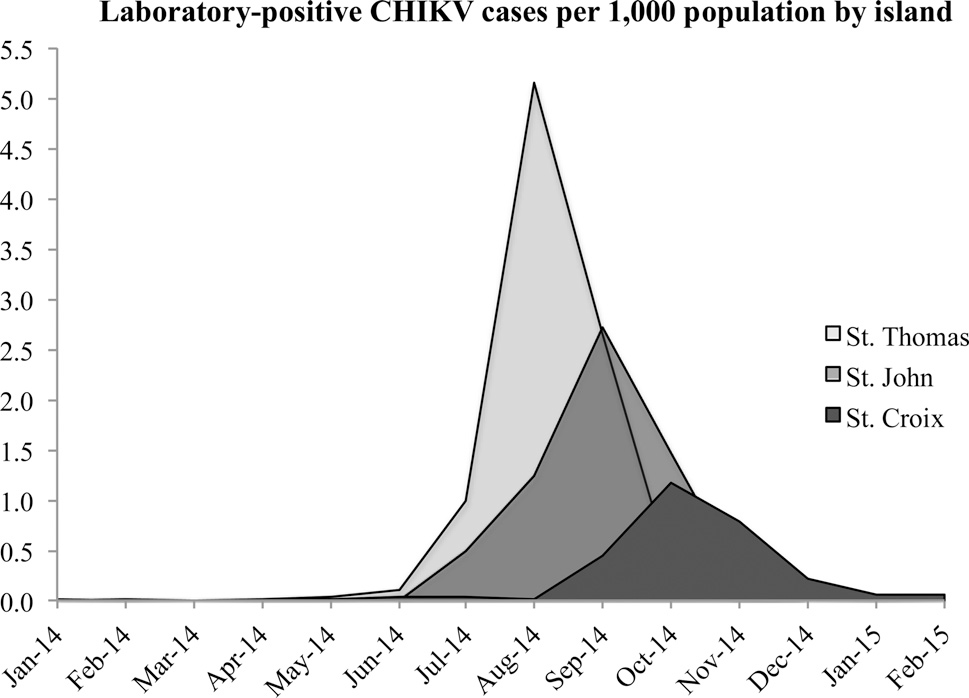
Epidemic curve of reported laboratory-positive chikungunya virus cases per 1,000 population by month of illness onset and island from January 2014 to February 2015.

A total of 1,929 suspected cases of CHIKV were reported to the USVI DOH between January 1, 2014, and April 6, 2015. Due to limited healthcare capacity and cost of laboratory testing, only 912 (47%) of the suspected cases had blood specimens that were tested for CHIKV. Of all suspected cases with a tested blood specimen, 275 (30%) were laboratory negative and 637 (70%) were laboratory positive (6.15 positive cases per 1,000 population during January 2014–April 2015). Of the laboratory-positive cases, 469 (74%) were residents living on Saint Thomas (9.33 positive cases per 1,000 population during January 2014–April 2015), 143 (22%) were living on Saint Croix (2.90 positive cases per 1,000 population during January 2014–April 2015) and 25 (4%) were living on Saint John (6.16 positive cases per 1,000 population during January 2014–April 2015). On the basis of the epidemic curve, Saint Thomas experienced the outbreak more severely and earlier in the year than the islands of Saint Croix and Saint John (Figure 1

). Peak incidence of reported laboratory-positive cases was 2.56 per 1,000 population and occurred during August 2014.

### Demographic characteristics.

Of those presenting at a hospital or healthcare clinic, the median age of laboratory-positive cases was 46 years, whereas the median age of laboratory-negative suspected cases was 41 years (Table 1). The mean difference in age between laboratory-positive cases and laboratory-negative suspected cases was 3.9 years (*P* value = 0.03). CHIKV incidence was highest among individuals aged 55–64 years and ≥ 65 years (13.06 and 11.71 cases per 1,000 population, respectively) and lowest among individuals aged 0–14 years and 25–54 years (1.77 and 2.39 cases per 1,000 population, respectively, Table 2). A larger percentage of laboratory-positive cases was female (60%) compared with male (40%), and this was consistent when stratifying by island. Overall, incidence was higher among females compared with males (6.57 and 5.00 cases per 1,000 population, respectively). CHIKV incidence, however, was slightly higher among males aged 0–24 years than females of the same age (Table 2). Laboratory-positive cases were 14% (95% confidence interval [CI] = 2–27%) more likely than laboratory-negative suspected cases to have contact with a household member who was recently ill (Table 1). After adjusting for age and gender, the percentage increased to 18% (95% CI = 5–32%). Traveling outside of the country 14 days before onset of illness was not associated with being a laboratory-positive case.

### Clinical manifestations.

A larger proportion of laboratory-positive cases had fever lasting 2–7 days, myalgia, headache, chills/rigor, anorexia, and were unable to walk compared with laboratory-negative suspected cases (Table 1). A larger proportion of laboratory-negative individuals had a sore throat, nasal congestion, cough, rash, and diarrhea. When examining all reported clinical manifestations together in a multivariate model, no symptoms apart from fever ≥ 38°C and arthralgia were associated with reported CHIKV infection. Only one symptom remained significantly associated with not being a case; laboratory-positive cases were 25% (95% CI = 4–41%) less likely to have diarrhea compared with laboratory-negative suspected cases.

## Discussion

In 2014, the USVI was one of many island regions in the Caribbean to experience the first documented CHIKV outbreak in the Americas. A total of 1,929 suspected cases were reported to the USVI DOH. Although the last laboratory-positive case of CHIKV in the USVI was reported in February 2015, it is unclear whether CHIKV transmission will reoccur in subsequent years. Reemergence is of particular concern, given that CHIKV transmission is still ongoing in many neighboring countries and could become endemic in the region along with other important arboviruses such as Zika and dengue. It is therefore imperative to learn from the 2014 outbreak to enhance early surveillance efforts and strengthen public health prevention methods against arboviral diseases.

Despite similar-sized populations, Saint Thomas had a larger proportion of CHIKV cases than Saint Croix, likely in part due to the higher population density (1,649.1 compared with 607.3 persons per square mile, respectively). The larger proportion of cases may also be due to the fact that Saint Thomas received almost three times the number of air passenger arrivals and almost 16 times more cruise ship passengers than Saint Croix in 2014.^14^,^15^ The relative hypermobility of the Saint Thomas population as well as the increased population density due to both visitors and residents may have helped facilitate the spread and reintroduction of CHIKV.^21^

Overall, laboratory-positive cases were older than laboratory-negative suspected cases. Individuals aged 55 years or more had the highest reported CHIKV incidence, which is consistent with findings from previous outbreaks where increased age was associated with symptomatic infection and severe atypical disease.^6^,^22^–^24^ Older individuals may have been more likely to seek health care for CHIKV infection and more likely to have experienced symptomatic or severe disease than younger individuals.

Aside from having fever ≥ 38°C and arthralgia or arthritis, no other clinical symptoms were significantly associated with CHIKV infection. Clinical manifestations of laboratory-positive cases from the USVI outbreak were consistent with symptoms reported in prior outbreaks among confirmed cases in Singapore, India, Malaysia, La Réunion, and Suriname.^7^,^18^,^23^,^25^–^28^ Of note, a larger proportion of laboratory-positive cases in the USVI reported myalgia (93%) and eye pain (35%) compared with cases from previous outbreaks in other regions of the world.^7^,^18^,^23^,^25^–^28^

Contact with a recently ill household member was associated with being a laboratory-positive case. This is typical for diseases spread by *Ae. aegypti* and *Ae. albopictus* mosquitoes which tend to be domestic/peridomestic in nature with limited flight ranges (78–230 m).^29^,^30^ Mosquitoes breeding near one household are capable of infecting persons living within a certain distance of that house, and the greater the number of people living within that range, the greater the opportunity the mosquito has to transmit CHIKV to a human. It is therefore not surprising to observe an increased risk of being a case given contact with a previously ill household member.^31^

Several limitations of this study should be highlighted when considering the results. The sample was one of convenience because only cases who sought health care for their symptoms were included in the analysis. As a result, our analysis likely overrepresented CHIKV cases presenting with severe disease because they were more likely to seek health care and receive laboratory testing than nonsevere cases. In addition, the quality of the surveillance data was dependent on the providers' ability to consistently and accurately report suspected cases and their clinical symptoms. Although providers were educated on the importance of capturing this data, monitoring of the reporting was not conducted. Laboratory-negative suspected cases may not be the optimal comparison group for this analysis, because although they are similar to cases in regard to healthcare seeking behavior, they may not be representative of the larger USVI population. Lastly, only 47% of suspected reported cases received laboratory testing for CHIKV, either because the healthcare facilities ran out of resources to continue laboratory testing or because suspected cases refused to be laboratory tested. Refusal was likely due to the cost of the test or fear of needles. Because the sample tested was not a random sample of all suspected cases, the results may not accurately represent the demographic characteristics of the CHIKV outbreak in the USVI and the true incidence of CHIKV remains unknown. Large-scale serological studies capable of detecting the seroconversion rates of these populations will be useful in capturing true incidence.

A variety of other factors including human mobility/behavior, population density, herd immunity, mosquito abundance, climate, and socioeconomic conditions are responsible for the CHIKV patterns observed in the USVI and Caribbean.^32^ A more detailed understanding of the true incidence and recent epidemic dynamics will be valuable in understanding differences in morbidity between countries, prediction of future outbreaks, and potential consequences of human-driven change including urbanization, globalization, and climate change.

Despite certain limitations, the present investigation describes the clinical manifestations associated with the first CHIKV outbreak in the USVI and identifies the most vulnerable populations for CHIKV disease. These results contribute to our knowledge of CHIKV disease and may aid in mitigating future CHIKV outbreaks in the Caribbean.

## Figures and Tables

**Table 1:** Proportion of suspected (but not tested), laboratory-positive and negative CHIKV cases by demographic risk factors and clinical manifestations, as well as univariate prevalence ratio estimates comparing laboratory-positive cases and laboratory-negative suspected cases

Demographic risk factor/clinical manifestation	Suspected (*n*)*	Positive (*n*)	Negative (*n*)	Prevalence ratio (95% confidence interval)
Median age (years)	43.25 (880)	45.99 (572)	41.04 (248)	–
Female	0.58 (551)	0.60 (364)	0.63 (165)	0.96 (0.88–1.05)
Traveled 14 days before illness onset	0.05 (35)	0.07 (32)	0.11 (21)	0.83 (0.66–1.04)
Contact with recently ill household member	0.25 (166)	0.25 (103)	0.17 (29)	**1.14 (1.02–1.27)**
Pregnant	0.02 (5)	0.06 (12)	0.02 (1)	**1.27 (1.06–1.51)**
Fever ≥ 38°C†	0.65 (460)	0.77 (404)	0.69 (152)	**1.14 (1.01–1.28)**
Fever (2–7 days)	0.71 (476)	0.80 (362)	0.75 (148)	1.08 (0.95–1.24)
Arthralgia	0.94 (826)	0.94 (562)	0.86 (214)	**1.45 (1.14–1.84)**
Arthritis	0.43 (317)	0.44 (246)	0.40 (94)	1.06 (0.96–1.16)
Nausea/vomiting	0.24 (141)	0.21 (92)	0.25 (46)	0.93 (0.81–1.06)
Rash	0.42 (274)	0.33 (144)	0.39 (74)	0.93 (0.83–1.04)
Myalgia	0.86 (622)	0.93 (441)	0.84 (165)	**1.46 (1.13–1.88)**
Diarrhea	0.16 (92)	0.12 (54)	0.25 (48)	**0.72 (0.60–0.87)**
Fatigue/malaise	0.22 (118)	0.27 (117)	0.35 (64)	0.89 (0.79–1.00)
Headache	0.70 (458)	0.70 (316)	0.67 (130)	1.03 (0.92–1.15)
Chills/rigor	0.57 (335)	0.67 (299)	0.58 (108)	1.12 (1.00–1.25)
Eye pain	0.35 (197)	0.35 (140)	0.35 (62)	1.01 (0.90–1.13)
Anorexia	0.36 (180)	0.56 (231)	0.50 (88)	1.07 (0.96–1.20)
Unable to walk	0.39 (263)	0.44 (222)	0.34 (72)	**1.14 (1.03–1.25)**
Cough	0.16 (103)	0.11 (51)	0.19 (40)	0.78 (0.65–0.94)
Nasal congestion	0.13 (78)	0.07 (31)	0.14 (27)	0.74 (0.58–0.95)
Sore throat	0.15 (96)	0.10 (47)	0.16 (33)	0.83 (0.68–1.00)

CHIKV = chikungunya virus. Statistically significant univariate prevalence ratio estimates are in bold.

* Individuals suspected of CHIKV infection but without confirmed laboratory-test results.

† Fever ≥ 38°C was marked “yes” only if the individual was febrile at the time of medical visit.

**Table 2:** Chikungunya virus cases per 1,000 population by age category and gender from January 2014 to February 2015^13^

	Cases/1,000 population		
Age category	Males	Females	Total
0–14	1.75	1.67	1.77
15–24	8.62	5.20	7.55
25–54	1.33	3.18	2.39
55–64	10.01	14.58	13.06
≥ 65	9.77	12.31	11.71
Total	5.00	6.57	6.15
